# Creation of Philadelphia chromosome by CRISPR/Cas9-mediated double cleavages on *BCR* and *ABL1* genes as a model for initial event in leukemogenesis

**DOI:** 10.1038/s41417-022-00522-w

**Published:** 2022-08-23

**Authors:** Minori Tamai, Shinichi Fujisawa, Thao T. T. Nguyen, Chiaki Komatsu, Keiko Kagami, Kenji Kamimoto, Kohei Omachi, Shin Kasai, Daisuke Harama, Atsushi Watanabe, Koshi Akahane, Kumiko Goi, Kazuhito Naka, Tadashi Kaname, Takanori Teshima, Takeshi Inukai

**Affiliations:** 1 Department of Pediatrics, School of Medicine, University of Yamanashi, Yamanashi, Japan; 2 Division of Laboratory and Transfusion Medicine, Hokkaido University Hospital, Hokkaido, Japan; 3 Department of Developmental Biology, Washington University School of Medicine in St. Louis, St Louis, MO USA; 4 Division of Nephrology, Washington University School of Medicine in St. Louis, St. Louis, MO USA; 5 Department of Stem Cell Biology, Research Institute for Radiation Biology and Medicine, Hiroshima University, Hiroshima, Japan; 6 Department of Genome Medicine, National Research Institute for Child Health and Development, Tokyo, Japan

**Keywords:** Leukaemia, Cancer genetics

## Abstract

The Philadelphia (Ph) chromosome was the first translocation identified in leukemia. It is supposed to be generated by aberrant ligation between two DNA double-strand breaks (DSBs) at the *BCR* gene located on chromosome 9q34 and the *ABL1* gene located on chromosome 22q11. Thus, mimicking the initiation process of translocation, we induced CRISPR/Cas9-mediated DSBs simultaneously at the breakpoints of the *BCR* and *ABL1* genes in a granulocyte-macrophage colony-stimulating factor (GM-CSF) dependent human leukemia cell line. After transfection of two single guide RNAs (sgRNAs) targeting intron 13 of the *BCR* gene and intron 1 of the *ABL1* gene, a factor-independent subline was obtained. In the subline, p210 *BCR::ABL1* and its reciprocal *ABL1::BCR* fusions were generated as a result of balanced translocation corresponding to the Ph chromosome. Another set of sgRNAs targeting intron 1 of the *BCR* gene and intron 1 of the *ABL1* gene induced a factor-independent subline expressing p190 *BCR::ABL1*. Both p210 and p190 BCR::ABL1 induced factor-independent growth by constitutively activating intracellular signaling pathways for transcriptional regulation of cell cycle progression and cell survival that are usually regulated by GM-CSF. These observations suggested that simultaneous DSBs at the *BCR* and *ABL1* gene breakpoints are initiation events for oncogenesis in Ph+ leukemia. (200/200 words).

## Introduction

Chromosomal translocation is among the most common chromosomal abnormalities observed in leukemia, and is highly involved in leukemogenesis. It is supposed to be generated as a result of aberrant repairs of two simultaneous DNA double-strand breaks (DSBs) at different portions of the chromosome. The Philadelphia (Ph) chromosome was the first chromosomal translocation identified in cancer. It was discovered in 1960 by Nowell PC and Hungerford DA as an abnormal minute chromosome in chronic myeloid leukemia (CML) patients [1]. In 1973, using chromosome banding techniques, Rowley JD demonstrated that it is a balanced translocation between chromosomes 22 and 9 [2]. It was also identified in acute lymphoblastic leukemia (ALL) [3]. Later studies identified the *BCR* gene and the *ABL1* gene at each breakpoint [4, 5]. There are two major types of Ph chromosome. In most CML patients and approximately one-quarter of Ph chromosome-positive (Ph + ) ALL patients, exon 13 or 14 of the *BCR* gene is fused to exon 2 of the *ABL1* gene, which encodes p210 BCR::ABL1 fusion protein [6]. In the other Ph+ ALL patients, exon 2 of the *BCR* gene is fused to exon 2 of the *ABL1* gene, which encodes p190 BCR::ABL1 fusion protein [7]. In BCR::ABL1 oncoprotein, the tyrosine kinase domain of ABL1 is constitutively activated due to acquisition of a dimerization domain of BCR and a loss of the SH3 domain of ABL1, which negatively regulates ABL1 kinase activity [8]. BCR::ABL1 potently activates diverse signaling pathways involved in leukemic transformation by promoting cell cycle progression and cell survival [9–12]. Of clinical importance, tyrosine kinase activity of BCR::ABL1 protein is proven to be an effective therapeutic target [13]. Tyrosine kinase inhibitors (TKIs) have dramatically improved prognoses of CML and Ph+ ALL patients [14–19].

For functional evaluation of tyrosine kinase activity of BCR::ABL1 and pharmacogenetic evaluation of *BCR::ABL1* gene mutations on TKI sensitivities, a murine IL-3-dependent Baf3 cell line transduced with human *BCR::ABL1* cDNA by retrovirus vector has been generally used [20, 21]. A bona fide model of CML was initially developed in lethally irradiated mice after syngeneic transplantation of bone marrow, in which *BCR::ABL1* cDNA was retrovirally transduced [22]. Although leukemia progression was not achieved by simple transplantation of human CD34 + cord blood cells retrovirally transduced with p210 *BCR::ABL1* cDNA into NOD-SCID mice [23], simultaneous transduction of *BMI1* cDNA induced ALL progression [24]. In transgenic mice of p210 or p190 *BCR::ABL1* under diverse promoters, leukemia progression has been widely confirmed [25–27]. The leukemogenic potential of *BCR::ABL1* was also evaluated in the endogenous locus of mouse *bcr* gene promoter. Notably, B-cell leukemia was developed in knock-in mice of p190 *BCR::ABL1* cDNA [28], while leukemia progression was not confirmed in those of p210 *BCR::ABL1* [29].

One possible explanation for this discrepancy between transgenic models and knock-in models is that promoter activity of the *bcr* gene in the knock-in mice might not be sufficiently high for leukemia transformation by p210 *BCR::ABL1*. In this context, 3’-untranslated region (UTR) is also involved in transcriptional and post-transcriptional regulation [30, 31]. In the *BCR::ABL1* gene, the involvement of microRNA in post-transcriptional regulation through 3’ UTR has been reported [32]. However, 3’ UTR of the *BCR::ABL1* cDNA was largely deleted in the above mice models. Moreover, cDNA lacks introns. Although its significance in leukemogenesis remains to be elucidated, alternative splicing of the *BCR::ABL1* gene has been reported to be involved in TKI resistance [33]. Another difference between human Ph+ leukemia and the above mice models is reciprocal *ABL1::BCR* fusion derived from balanced translocation. Gene transfer of reciprocal *ABL1::BCR* fusion into murine hematopoietic stem cells enhanced proliferation and stem cell capacity of early progenitors [34], suggesting the involvement of reciprocal *ABL1::BCR* in leukemogenesis. Under these circumstances, the development of a novel platform that permits testing of leukemogenic activities of balanced translocation under intrinsic transcriptional and post-transcriptional regulation is indispensable.

In the present study, we sought to investigate the hypothesis that the Ph chromosome is generated by aberrant repair of two simultaneous DSBs at the *BCR* and *ABL1* gene breakpoints as an initiation event for leukemogenesis. Thus, we induced DSBs at specific breakpoints of *BCR* and *ABL1* genes, using the CRISPR/Cas9 system in a human factor-dependent leukemia cell line. The obtained factor-independent sublines acquired p210 or p190 *BCR::ABL1* and their reciprocal fusion genes as a result of balanced translocation, which is cytogenetically identical to the Ph chromosome. Using these sublines, we evaluated the significance of p210 and p190 *BCR::ABL1* in signal transduction and transcription profile.

## Methods

### Creation of *BCR::ABL1* fusion by CRISPR/Cas9

Synthesized self-complementary oligomers designed by Benchling software (https://www.benchling.com) (Supplement Table 1) and ligation adaptors were purchased from IDT (https://sg.idtdna.com). Each single guide RNA (sgRNA) and pSpCas9(BB)-2A-GFP (Addgene, Watertown, MA, #48138) was amplified by polymerase chain reaction (PCR) and ligated using a NEBuilder HiFi DNA assembly kit (New England BioLabs, Ipswich, MA, USA). A 293 T cell line was maintained with 10% fetal calf serum (FCS) containing Dulbecco’s modified Eagle’s medium (DMEM) medium. The plasmid was transfected using Lipofectamine 3000 (Thermo Fisher Scientific, Waltham, MA, USA). A TF-1 cell line was purchased from ATCC (#CRL-2003, Manassas, VA, USA) and expanded with 10% FCS containing RPMI1640 medium with 2 ng/ml of human recombinant granulocyte-macrophage colony-stimulating factor (GM-CSF) (PeproTech, Cranbury, NJ, USA). The plasmid was transfected using the Neon electroporation system (Thermo Fisher Scientific) with single pulse at 1300 volts for 20 msec. The cells were cultured in the presence of GM-CSF for seven days. Subsequently, 1 × 10^4^ cells were placed in a 24-well plate in the absence of GM-CSF. The number of living cells was counted every seven days after trypan blue staining.

### Polymerase chain reaction (PCR) analyses

Genomic DNA was extracted using a PureLink Genomic DNA Mini Kit (Thermo Fisher Scientific). The sequence of each primer is listed in Supplement Table 2. PCR products were subcloned using a TA Cloning Kit (Thermo Fisher Scientific) and directly sequenced using each forward primer. For PCR analysis of the *BCR::ABL1* transcript, total RNAs were extracted using a RNeasy Plus Mini Kit (QIAGEN, Hilden, Germany), and complementary DNAs (cDNAs) were generated with SuperScript IV Reverse Transcriptase (Thermo Fisher Scientific). Amplification was performed using the primers listed in Supplement Table 2.

### Short tandem repeat (STR) analysis

Genomic DNA was extracted using a QIAamp DNA Blood Mini Kit (QIAGEN). PCR was performed using the fluorescent primers listed in Supplement Table 3. The PCR products were analyzed using an ABI 3500 Genetic Analyzer system (Thermo Fisher Scientific) and quantified using GeneMapper software, v4.1 (Thermo Fisher Scientific).

### G-band karyotyping and fluorescence in situ hybridization (FISH)

After 2 h of treatment with 0.1 ug/ml of KaryoMAX COLCEMID Solution (Thermo Fisher Scientific), the cells were exposed to 0.075 mol/l of KCL at 37 °C for 15 min and fixed on slide glasses, using a 3:1 methanol/glacial acetic acid solution three times. After trypsin-Giemsa staining of the air-dried slide samples, 20 metaphases were analyzed for each sample. For FISH analysis, the air-dried slide samples were denatured at 75 °C for 1 min and hybridized with LSI^TM^ BCR Dual Fusion and LSI^TM^ ASS-ABL probes (Vysis/Abbott, Abbott Park, IL, USA) at 37 °C for 50 h. Karyotypic and FISH analyses were performed using a CytoVision system (Applied Imaging, Santa Clara, CA, USA).

### Spectral karyotyping (SKY)

Spectral karyotyping (SKY) was performed with a SkyPaint probe mixture (Applied Spectral Imaging, Migdal HaEmek, Israel). Briefly: after denaturation, each sample was hybridized at 37 °C for 67 hr and counterstained with DAPI I (4′6-diamino-2-phenylindole, dihydrochloride, Fa. Vysis). Karyotyping was performed using a SpectraCube system with SkyView 1.5 (Applied Spectral Imaging).

### Cell cycle and cell apoptosis analysis

Cells were stained with propidium iodide (PI) (Sigma, St. Louis, MO, USA) or fluorescein isothiocyanate (FITC) conjugated with Annexin V and PI (MBL, Nagoya, Japan). Each sample was analyzed using flow cytometry (FACSCelesta, BD Biosciences, Franklin Lakes, NJ, USA). Data evaluation was performed using FACS Diva software (v.8.0.1.1, BD Biosciences) and FlowJo software (v.10.6.1, LLC, Ashland, OR, USA).

### AlamarBlue assay

Cells (0.1 × 10^4^) were incubated with six concentrations of imatinib, dasatinib, nilotinib, or ponatinib, in triplicate, using a 96-well plate. After 66 hr of incubation, the cells were additionally incubated with alamarBlue (Bio-Rad Laboratories, Hercules, CA, USA) for 6 h [35]. Absorbance at 570 nm was monitored by spectrophotometer, using 600 nm as a reference wavelength. Cell viability was calculated by the ratio of the optical density of the treated wells to that of the untreated wells, as a percentage.

### Western blot analyses

Cells were treated with 1 mM of 4-(2-aminoethyl) benzene sulfonyl fluoride HCl (Calbiochem, Darmstadt, Germany) on ice for 10 min, then solubilized in lysis buffer (50 mM Tris-HCl, pH 7.5, 150 mM NaCl, 1% Nonidet P-40, 5 mM EDTA, 0.05% NaN3, 1 mM phenylmethylsulfonyl fluoride, 100 μM sodium vanadate). The lysates were separated on a SDS-polyacrylamide gel under reducing conditions and transferred to a nitro-cellulose membrane. The membrane was incubated overnight at 4 °C with the primary antibodies listed in Supplement Table 4, and subsequently with a horseradish peroxidase-labeled second antibody (MBL) at room temperature for 1 h, and developed using an ECL Prime Western Blotting Detection Kit (GE Healthcare, Little Chalfont, UK).

### Quantitative real-time (RT) PCR

Triplicated samples with SYBR Green PCR Master Mix (Thermo Fisher Scientific) were amplified through 40 cycles (at 95 °C for 15 sec and 60 °C for 1 min), using the primers listed in Supplement Table 5. Quantitation was performed using an ABI Prism 7500 Sequence Detection System (Thermo Fisher Scientific). The relative gene expression level was determined using *INHβB* as an internal control.

### mRNA sequencing analysis

Total RNA was extracted from the triplicated samples using a RNeasy Plus Mini Kit (QIAGEN). The library preparation and sequencing were entrusted to Bioengineering Lab (Sagamihara, Japan). RNA concentration and quality were evaluated with a Quantus Fluorometer, using the QuantiFluor RNA system (Promega, Madison, WI, USA), and a 5200 Fragment Analyzer System, using an Agilent HS RNA Kit (Agilent Technologies, Santa Clara, CA, USA). A sequence library was prepared, using an MGIEasy RNA Directional Library Prep Set (MGI Tech, Kobe, Japan). Library concentration and quality were evaluated with a Qubit 3.0 Fluorometer, using a dsDNA HS Assay Kit (Thermo Fisher Scientific), and with a Fragment Analyzer, using a dsDNA 915 Reagent Kit (Agilent Technologies). Construction of cDNA was performed using an MGIEasy Circularization Kit (MGI Tech). The DNA nanoballs generated with a DNBSEQ-G400RS High-throughput Sequencing Kit (MGI Tech) were sequenced using DNBSEQ-G400 (2 × 100 bp). Adaptor sequences, indexes, and primer regions were removed, using Cutadapt (v.1.9.91). Read sequences with low-quality scores (<20) and short read lengths (<40 bp) were removed, using Sickle (v.1.33). The read sequences were aligned to the human reference genome (GRCh38.p13; https://www.ncbi.nlm.nih.gov/assembly/GCF_000001405.39), using hisat2 (v.2.2.1). Reads were counted and normalized using featureCount (v.2.0.0). The total number of reads and gene lengths among samples were corrected, using transcripts per million (TPM). Differentially expressed genes among samples were analyzed using a DEseq2 package (v.1.30.1) [36]. Principal component analysis was performed using pcaExplorer (v.2.21.0) and scatterplot3d (v.0.3-41) packages [37]. A heatmap and a volcano plot were generated using ComplexHeatmap (v.2.9.4) [38] and EnhancedVolcano (v.1.8.0) [39], respectively. Gene ontology (GO) analysis and gene set enrichment analysis (GSEA) were performed using Metascape [40] and GSEA software (v.4.1.0) [41], respectively.

## Results

### Creation of p210 *BCR::ABL1* fusion gene by double cleavages of *BCR* and *ABL1* genes, using CRISPR/Cas9

**Fig. 1:**
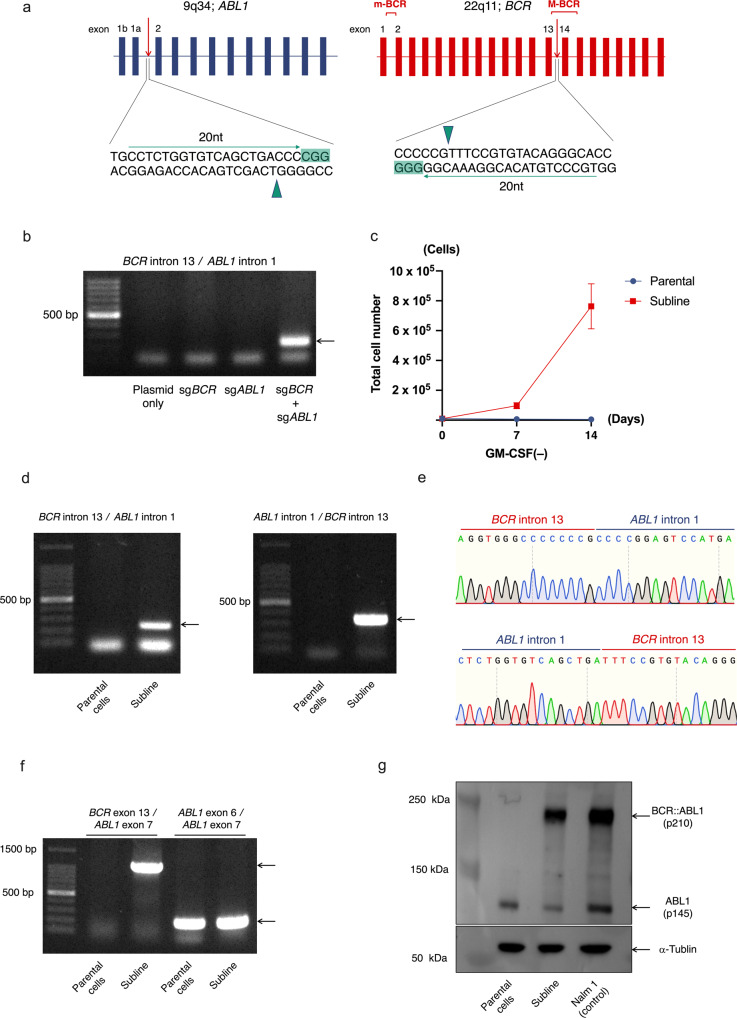
Creation of p210 *BCR::ABL1* fusion gene, using the CRISPR/Cas9 system. **a** Schematic representation of sgRNA target sites. Targeted protospacer adjacent motif (PAM) site is highlighted in orange. Arrows and arrowheads indicate sequences of sgRNA and Cas9 cleavage sites, respectively. **b** Genomic PCR of *BCR::ABL1* junctional region in 293 T cell lines transfected with either or both of two sgRNAs for the *BCR* and *ABL1* genes. **c** Growth curves of parental TF-1 cells and subline cultured in the absence of GM-CSF, with error bars of triplicated samples. **d** Genomic PCR of *BCR::ABL1* and *ABL1::BCR* junctional regions in parental cells and subline. **e** Representative genomic sequences of *BCR::ABL1* (top panel) and reciprocal *ABL1::BCR* (bottom panel) fusion sites. **f** RT-PCR analysis of the *ABL1* and p210 *BCR::ABL1* genes in parental cells and subline. Genes with exon numbering of forward and reverse primers are indicated (top of panel). **g** Western blot analysis of parental cells and subline with anti-ABL1 and anti-α-tubulin antibodies using Nalm1 cell line as a positive control.

In CML, a p210 *BCR::ABL1* fusion gene is generated between exon e13 of the *BCR* gene and exon a2 of the *ABL1* gene. To generate e13a2 type fusion, we transfected two sgRNAs targeting intron 13 of the *BCR* gene and intron 1 of the *ABL1* gene (Fig. 1a) with *Cas9* cDNA into HEK293T, a human embryonic kidney cell line. Genomic PCR revealed formation of e13a2 type fusion when both sgRNAs were transfected (Fig. 1b). We next tried to create e13a2 type fusion in a TF-1 cell line, which is a human GM-CSF-dependent erythroleukemia cell line, since transfection of *BCR*::*ABL1* fusion cDNA was reported to induce factor-independent cell growth [42]. After transfection of two sgRNAs with *Cas9* cDNA, TF-1 cells were first expanded in the presence of GM-CSF for seven days, and subsequently cultured in the absence of GM-CSF. Seven days after GM-CSF depletion, the factor-independent cells started to expand. The obtained subline proliferated without GM-CSF (Fig. 1c), while parental cells were unable to grow without GM-CSF. Genomic PCR revealed generation of e13a2 and reciprocal a2e13 fusions in the subline (Fig. 1d). Sanger sequencing after TA-cloning confirmed direct ligations of two target sites of the sgRNAs in the majority of both PCR products (Fig. 1e, Supplement Fig. 1a, b). RT-PCR analysis confirmed expression of the e13a2 fusion transcript in the subline but not in the parental cells (Fig. 1f). Consistently, Western blot analysis using anti-ABL1 antibody confirmed generation of aberrant protein in the subline, which showed an identical migration pattern and similar intensity to that of p210 BCR::ABL1 fusion protein in a Nalm1 cell line (Fig. 1g). Finally, STR analysis showed an identical pattern between parental cells and subline (Supplement Fig. 2), thus excluding contamination of other cells. These results indicate the generation of the p210 *BCR::ABL1* fusion gene as a result of balanced translocation by direct ligation of two cleavage sites in a human leukemia cell line.

### Generation of balanced translocation corresponding to Ph chromosome

**Fig. 2:**
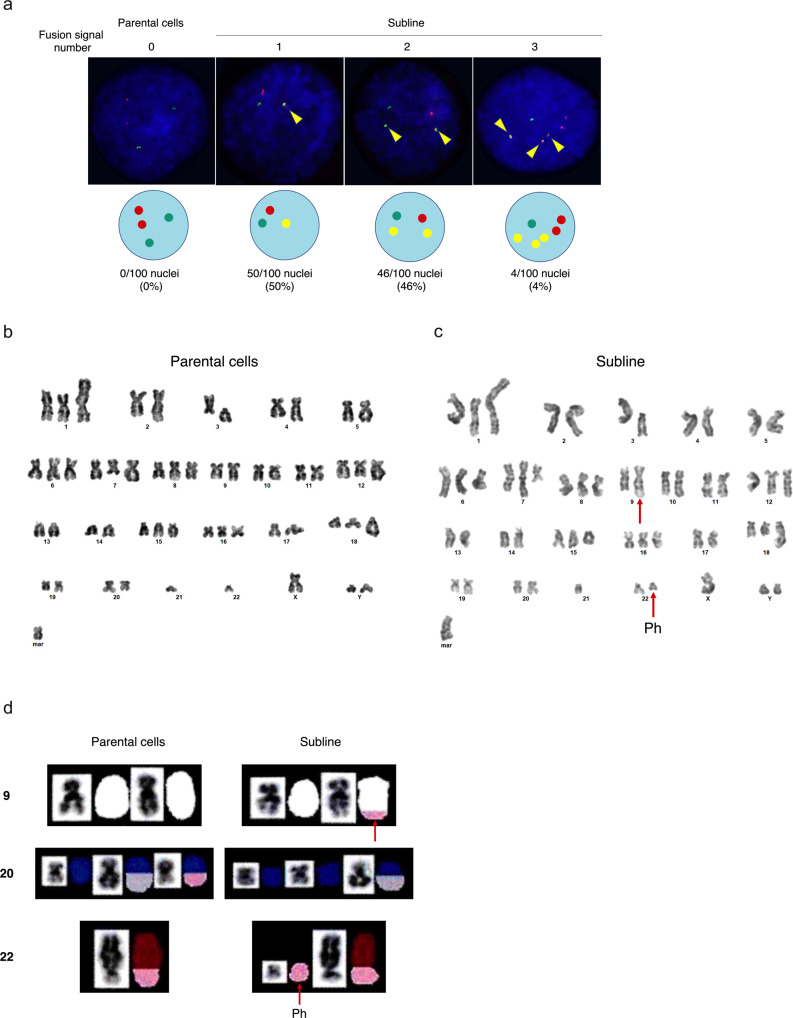
Cytogenetic analysis of p210 *BCR::ABL1* fusion gene. **a** Interphase FISH analysis of parental cells and subline, using a red probe for the *ABL1* gene and a green probe for the *BCR* gene. Merged yellow signals for *BCR*::*ABL1* and/or reciprocal *ABL1::BCR* fusion genes are indicated by arrow heads. **b**, **c** Representative G-banded karyotypes of parental cells **b** and subline **c**. Arrows indicate Ph chromosome and its balanced translocation. **d** Representative images of SKY analysis (chromosomes 9, 20, and 22) of parental cells and subline. Arrows indicate Ph chromosome and its balanced translocation.

In order to validate the artificial generation of a p210 *BCR::ABL1* fusion gene at chromosomal level, we first performed a FISH analysis (Supplement Fig. 3). In parental cells, two red signals corresponding to the *ABL1* gene and two green signals corresponding to the *BCR* gene were detectable in all nuclei. Notably, in the subline, single, double, and triple yellow fusion signals were detectable in 50%, 46%, and 4% of the nuclei, respectively (Fig. 2a, Supplement Fig. 4). In a G-banding analysis, parental cells showed highly rearranged hyperdiploidy with diverse variations (Fig. 2b). The subline showed similar structural and numerical abnormalities (Fig. 2c). Notably, the subline acquired balanced translocation resembling that of the Ph chromosome. To confirm the structural abnormality, we performed a SKY analysis. In the parental cells, chromosome 22 was translocated to chromosome 20. In the subline, the telomeric end of chromosome 22q was translocated to the centromeric end of chromosome 9q, and vice versa (Fig. 2d). These results indicate that double cleavages at the breakpoints of the p210 *BCR*::*ABL1* fusion gene by CRISPR/Cas9 artificially created a balanced translocation corresponding to the Ph chromosome.

### Creation of p190 *BCR::ABL1* fusion gene by double cleavages of *BCR* and *ABL1* genes, using CRISPR/Cas9

**Fig. 3:**
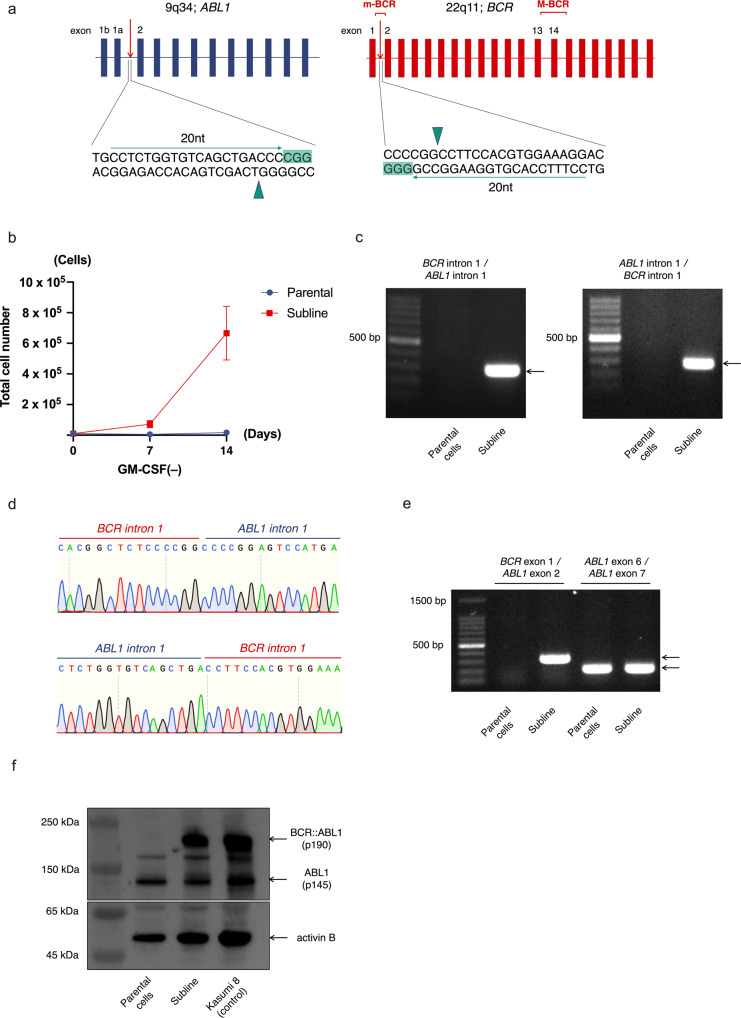
Creation of p190 *BCR::ABL1* fusion gene, using the CRISPR/Cas9 system. **a** Schematic representation of sgRNA target sites. **b** Growth curves of parental TF-1 cells and subline cultured in the absence of GM-CSF. **c** Genomic PCR of *BCR::ABL1* and *ABL1::BCR* junctional regions in parental cells and subline. **d** Representative genomic sequences of *BCR::ABL1* (top panel) and reciprocal *ABL1::BCR* (bottom panel) fusion sites. **e** RT-PCR analysis of the *ABL1* and p190 *BCR::ABL1* genes in parental cells and subline. **f** Western blot analysis of parental cells and sublines with anti-ABL1 and anti-activin-B antibodies, using Kasumi8 cell line as a positive control.

In most cases of Ph+ ALL, a p190 *BCR::ABL1* fusion gene is generated between exon e1 of the *BCR* gene and exon a2 of the *ABL1* gene. In order to create e1a2 type fusion, we transfected another set of sgRNAs targeting intron 1 of the *BCR* gene and intron 1 of the *ABL1* gene into a TF-1 cell line with *Cas9* cDNA (Fig. 3a). After selection in the absence of GM-CSF, we obtained a GM-CSF-independent subline (Fig. 3b). Genomic PCR analysis revealed generation of e1a2 and reciprocal a2e1 fusions in the subline (Fig. 3c). Sanger sequencing after TA-cloning revealed direct ligation at two breakpoints, with some minor variations in both genomic PCR products (Fig. 3d and Supplement Fig. 5a, b). RT-PCR analysis confirmed expression of the e1a2 fusion transcript in the subline but not in the parental cells (Fig. 3e). Western blot analysis of the subline using anti-ABL1 antibody revealed aberrant protein with an identical migration pattern and similar intensity to that of p190 BCR::ABL1 fusion protein in a Kasumi8 cell line (Fig. 3f). These results indicate the generation of a p190 *BCR::ABL1* fusion gene as a result of balanced translocation.

### Constitutive activation of artificially generated p210 and p190 BCR::ABL1 tyrosine kinases in TF-1 cells

**Fig. 4:**
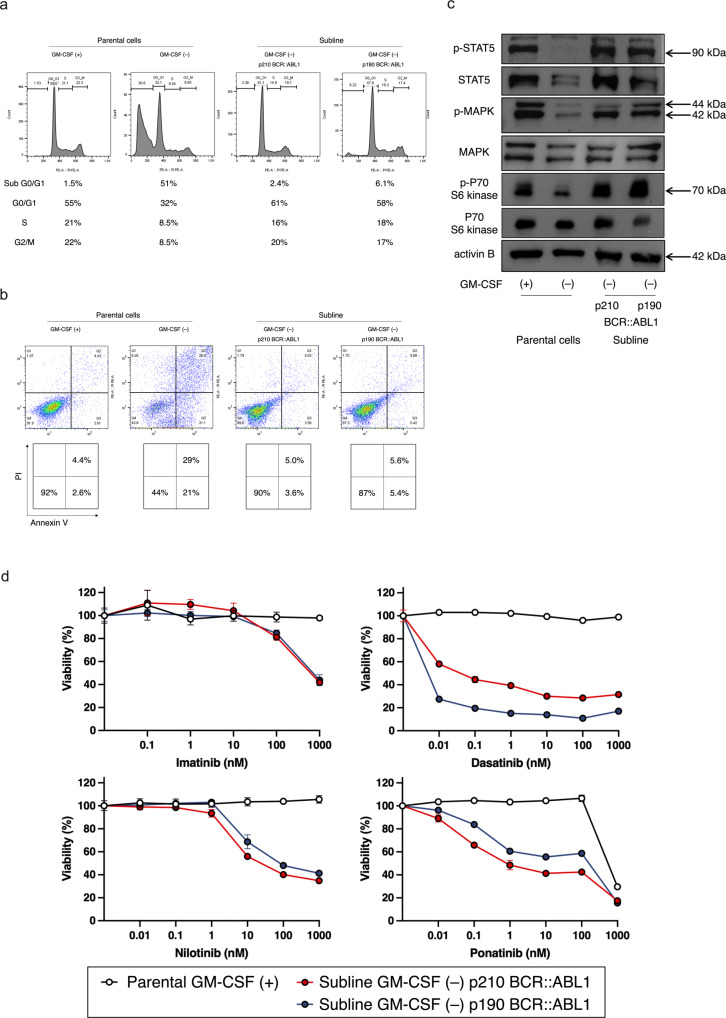
p210 and p190 BCR::ABL1 tyrosine kinase activities. **a** Cell cycle analysis of parental cells cultured in the presence or absence of GM-CSF and the p210 BCR::ABL1 and p190 BCR::ABL1 sublines cultured in the absence of GM-CSF. Percentages of sub-G0/G1, G0/G1, S, and G2/M phases are indicated at the bottom of each panel. **b** Apoptotic cell analysis of parental cells cultured in the presence or absence of GM-CSF and two sublines cultured in the absence of GM-CSF. Percentages of living, early apoptotic, and late apoptotic cells are indicated in each lower panel. **c** Phosphorylation of STAT5, MAPK, and P70/S6K in parental cells cultured in the presence or absence of GM-CSF and two sublines cultured in the absence of GM-CSF. **d** Dose–response curves of TKIs (imatinib, dasatinib, nilotinib, and ponatinib) in parental cells (white) cultured in the presence of GM-CSF, and the p210 BCR::ABL1 (red) and p190 BCR::ABL1 (blue) sublines cultured in the absence of GM-CSF.

Since two sublines showed GM-CSF-independent cell growth, we next evaluated the functional significance of BCR::ABL1 fusion proteins. In a cell cycle analysis, almost half of the parental cells cultured in the absence of GM-CSF (GM-) were accumulated in the sub-G0/G1 phase, while two GM- sublines showed almost similar distributions to those of the parental cells cultured in the presence of GM-CSF (GM + ) (Fig. 4a). In an apoptosis analysis (Fig. 4b), nearly half of the GM- parental cells underwent apoptosis, while most of two GM- sublines survived. We next evaluated the phosphorylation status of intracellular signaling molecules by Western blot analysis. In the GM- parental cells, STAT5, MAPK, and P70/S6K were dephosphorylated (Fig. 4c). In contrast, in two GM- sublines, STAT5, MAPK, and P70/S6K were constitutively phosphorylated (Fig. 4c). These observations indicate that GM-CSF-independent proliferation and cell survival of the p210 BCR::ABL1 and p190 BCR::ABL1 sublines were sustained by constitutive phosphorylation of intracellular signaling molecules. Notably, two GM- sublines were sensitive to all four TKIs (imatinib, dasatinib, nilotinib, and ponatinib), while the GM + parental cells were highly resistant (Fig. 4d). These observations indicate that artificially generated p210 BCR::ABL1 and p190 BCR::ABL1 were constitutively active in TF-1 cells cultured in the absence of GM-CSF.

### Distinctive transcriptional profile between p210 BCR::ABL1 and p190 BCR::ABL1 in TF-1 cells

**Fig. 5:**
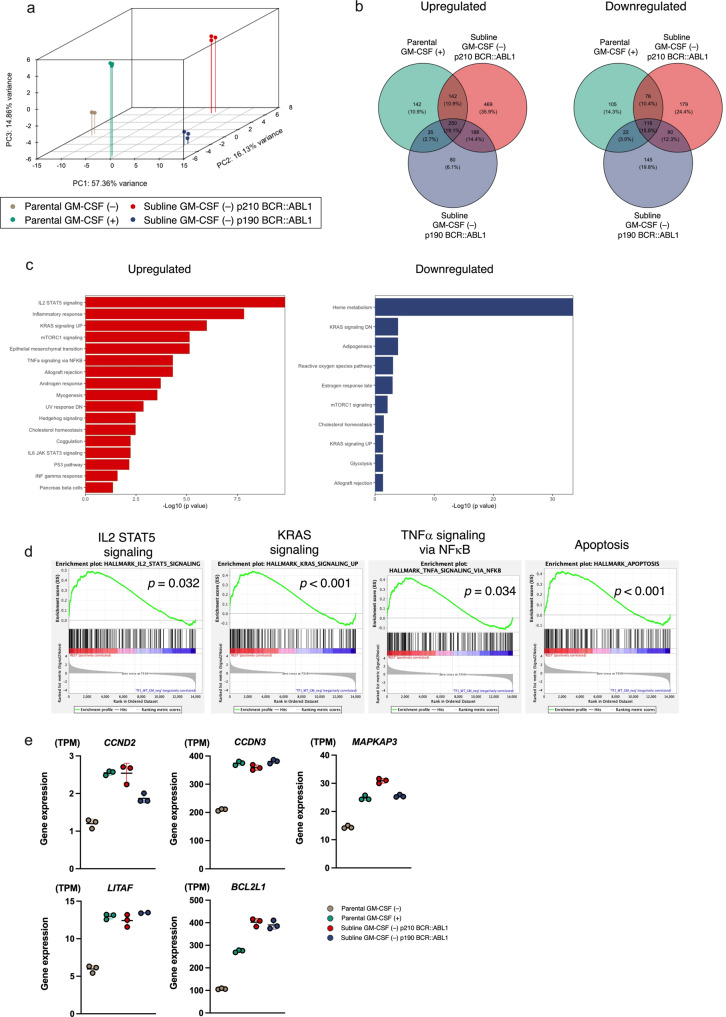
Transcriptional profiles in parental cells and BCR::ABL1 sublines. **a** 3D PCA analysis of transcriptional profile in parental cells cultured in the presence (green) or absence (gray) of GM-CSF, and the p210 BCR::ABL1 (red) and p190 BCR::ABL1(blue) sublines cultured in the absence of GM-CSF. **b** Venn diagram of commonly upregulated (left panel) and downregulated (right panel) genes (FDR < 0.01, Log2 fold change < 1 or > -1) in parental cells cultured in the presence of GM-CSF and two sublines cultured in the absence of GM-CSF, compared to the parental cells cultured in the absence of GM-CSF. **c** Gene ontology analysis of commonly upregulated (left panel) and downregulated (right panel) genes in the parental cells, cultured in the presence of GM-CSF, and two sublines cultured in the absence of GM-CSF, compared to the parental cells cultured in the absence of GM-CSF. **d** GSEA of common profile in the parental cells cultured in the presence of GM-CSF and two sublines cultured in the absence of GM-CSF, compared to the parental cells cultured in the absence of GM-CSF. **e** Gene expression levels of the *CCND2*, *CCND3*, *MAPKAPK3*, *LITAF*, and *BCL2L11* genes in triplicated samples of the parental cells cultured in the presence (green) or absence (gray) of GM-CSF and the p210 BCR::ABL1 (red) and p190 BCR::ABL1(blue) sublines cultured in the absence of GM-CSF.

Since artificially generated p210 BCR::ABL1 and p190 BCR::ABL1 are functionally active in TF-1 cells, we investigated the significance of transcriptional profile. RNA sequencing was performed in GM + and GM- parental cells and in two GM- sublines. In a principal component analysis, each sample clustered distinctly (Fig. 5a), and the gene expression profiles of the two GM- sublines were distinctly situated to each other. When compared with the GM- parental cells, the expression levels of 250 and 116 genes were commonly upregulated and downregulated, respectively, in the GM + parental cells and the two GM- sublines (Fig. 5b and Supplement Table 6). GO analysis indicated activation of STAT5 signaling, inflammatory response, and KRAS signaling, and inactivation of heme metabolism in the GM + parental cells and the two GM- sublines (Fig. 5c). In a GSEA, enrichment of STAT5 signaling, KRAS signaling, and TNFα signaling, and an apoptotic pathway were commonly observed in the GM + parental cells and the two GM- BCR::ABL1 sublines, compared to the GM- parental cells (Fig. 5d). Consistently, expression levels of the genes involved in cell cycle progression (*CCND2*, *CCND3*, and *MAPKAPK3*) and cell survival (*LITAF* and *BCL2L11*) were commonly upregulated in the GM + parental cells and the two GM- sublines (Fig. 5e and Supplement Fig. 6). These observations indicate that p210 and p190 BCR::ABL1 induced factor-independent cell growth by upregulating the genes involved in cell cycle progression and cell survival, which are normally regulated by growth factor stimulation.

**Fig. 6:**
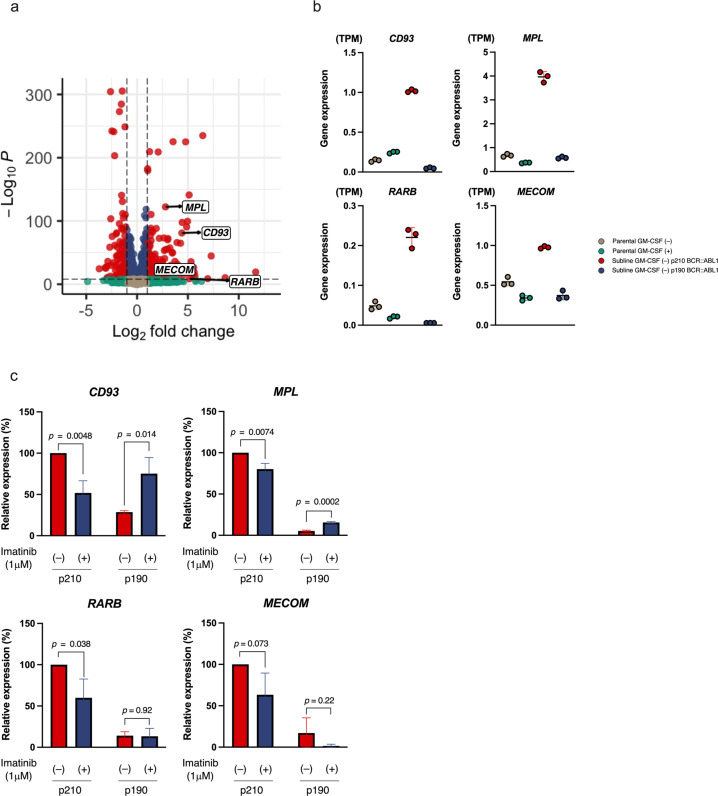
Distinct gene expression profile between p210 BCR::ABL1 and p190 BCR::ABL1 sublines. **a** Volcano plot of differentially expressed genes between the p210 BCR::ABL1 and p190 BCR::ABL sublines. Red plots indicate genes with *p*-value < 10^−9^ and absolute log2 fold change > 1. **b** Gene expression levels of the myeloid lineage-related genes (*CD93*, *MPL*, *RARB*, and *MECOM*) in triplicated samples of the parental cells cultured in the presence (green) or absence (gray) of GM-CSF, and the p210 BCR::ABL1 (red) and p190 BCR::ABL1(blue) sublines cultured in the absence of GM-CSF. **c** Effect of imatinib treatment on myeloid-related gene (*CD93*, *MPL*, *RARB*, and *MEIS1*) expression levels in triplicated samples of p210 BCR::ABL1 and p190 BCR::ABL sublines incubated with or without 1 μM of Imatinib for 24 h in the absence of GM-CSF. RT-PCR analyses were performed using *INHβB* as an internal control. Data are shown as mean ± standard deviation (SD). The *p*-values in a student *t*-test are indicated.

Considering the distinctive pattern in the principal component analysis, we concentrated on differences in gene expression profiles between the two GM- BCR::ABL1 sublines. Compared with the p190 BCR::ABL1 subline, 399 and 181 genes were upregulated and downregulated respectively in the p210 BCR::ABL1 subline (Fig. 6a). GO analysis revealed upregulation of extra-cellular matrix receptor interaction and hematopoietic lineage genes, and downregulation of systemic lupus erythematous genes in the p210 BCR::ABL1 subline, compared to the p190 BCR::ABL1 subline (Supplement Fig. 7). In a volcano plot analysis, myeloid lineage-related genes (*CD93*, *MPL*, *RARB*, and *MECOM*) were upregulated in the p210 BCR::ABL1 subline (Fig. 6b). We then evaluated changes in these myeloid lineage-related gene expression levels by real-time RT-PCR analysis in two GM- sublines after treatment with 1 μM of imatinib for 24 h. Notably, the gene expression levels of *CD93*, *MPL*, *RARB*, and *MECOM* were significantly downregulated by imatinib treatment in the p210 BCR::ABL1 subline, but were unchanged or upregulated in the p190 BCR::ABL1 subline (Fig. 6c). These observations indicate that p210 BCR::ABL1 specifically promotes myeloid features, compared to p190 BCR::ABL1.

## Discussion

Chromosomal translocation is generated by aberrant ligation of two simultaneous DSBs [43]. Interestingly, simultaneous DSBs on different chromosomes are reported to be sufficient to promote reciprocal translocations in mouse embryonic stem cell system [44]. When a genome editing system became available to induce a DSB of human genome at any locus of interest, *EWSR::FLI1* and *NPM1::ALK*, which are oncogenic fusions in Ewing sarcoma and anaplastic large cell lymphoma, respectively, were artificially generated in human mesenchymal precursors using zinc finger and transcription activator-like effector nucleases [45]. Subsequently, *EWSR::FLI1* and *NPM1::ALK* were generated by simultaneous cleavages at the target sites with the CRISPR/Cas9 system [46–49]. Similarly, leukemogenic *MLL* fusion genes including *MLL::AF4* [50–52], *MLL::AF9* [50, 53–55], and *MLL::ENL* [56] were generated by double cleavages using CRISPR/Cas9 in human hematopoietic cells and in the murine 32D myeloid progenitor cell line. For the development of incorrect ligation to take place between two DSBs, each DSB is supposed to be proximally located in the nucleus. Here, we note that the intergenic distance between *BCR* and *ABL1* genes in hematopoietic cells was reported to be less than expected [57, 58]. We hypothesized that simultaneous DSBs at two specific breakpoints of the *BCR* and *ABL1* genes by the CRISPR/Cas9 system may artificially generate a *BCR::ABL1* fusion gene in human cells as a result of balanced translocation. To test this hypothesis, we selected the human GM-CSF-dependent erythroleukemic cell line, TF-1, since generation of the *BCR::ABL1* fusion gene may induce factor-independent cell growth.

In the leukemogenesis of the Ph chromosome, the significance of the reciprocal *ABL1::BCR* fusion gene, generated as a result of balanced translocation, is controversial. In approximately one-third of CML cases, reciprocal *ABL1*::*BCR* fusion mRNA was undetectable [59], suggesting that the reciprocal *ABL1::BCR* fusion gene may not be indispensable, at least for development of CML. Meanwhile, gene transfer of the reciprocal *ABL1::BCR* fusion cDNA into murine hematopoietic stem cells enhanced proliferation and stem cell capacity of early progenitors [34]. Moreover, *ABL1::BCR* induced the B-cell commitment of murine hematopoietic stem cells and human umbilical cord blood cells. These in vitro models suggest that reciprocal *ABL1::BCR* might play a role in leukemogenesis by influencing the lineage commitment [34]. However, previous Ph+ leukemia models were unable to generate reciprocal *ABL1::BCR* fusion in combination with *BCR::ABL1* fusion. Notably, in the present study, genomic PCR analysis confirmed the generation of the reciprocal *ABL1::BCR* fusion gene and the *BCR::ABL1* fusion gene. In the FISH analysis of the GM-CSF-independent p210 *BCR::ABL1* subline, single and double fusion signals were observed in 50% and 46% of the nuclei, respectively. Accordingly, the cells with a single fusion signal were supposed to have the *BCR::ABL1* fusion gene only, while those with two fusion signals might have both the *BCR::ABL1* and the reciprocal *ABL1::BCR* fusion genes. Thus, at least half the population of the subline did not acquire or lost the reciprocal *ABL1::BCR* fusion gene during clonal evolution. These observations suggest that the reciprocal *ABL1::BCR* fusion gene is not indispensable, at least for factor-independent growth of TF-1 cells.

The genomic sequences at the *BCR::ABL1* and *ABL1::BCR* fusion sites of the p210 and p190 BCR::ABL1 sublines basically showed direct ligation of two cleavage sites, with minor variations. These direct ligations, without large insertion or deletion (indel), differed substantially from diverse large indels observed at the cleavage sites of genome editing with CRISPR/Cas9. These differences may be attributed to re-cleavage of the repair site by CRISPR/Cas9. In its usual repairing process by non-homologous end joining, a repair site might be repeatedly cleaved until massive indel is acquired [60]. In contrast, at the fusion site of two cleavage ends, further cleavage could not be induced, since the target sequences of sgRNAs are completely disrupted as a result of fusion [61].

Consistent with previous studies [62–65], we confirmed that both GM-CSF and BCR::ABL1 induced phosphorylation of intracellular signaling molecules, including STAT5, MAPK, and S6K. Moreover, the gene expression profile demonstrated that both GM-CSF and BCR::ABL1 upregulated a series of genes involved in cell cycle progression and cell survival. These observations indicate that p210 and p190 BCR::ABL1 induced factor-independent growth of TF-1 cells by constitutively activating intracellular signaling pathways for cell proliferation and cell survival, usually regulated by GM-CSF. Considering the factor-independent growth of the *BCR::ABL1* sublines, we compared the gene expression profile of the *BCR::ABL1* sublines and parental cells with that of 59 myeloid leukemia cell lines, including 13 Ph-positive cell lines (https://sites.broadinstitute.org/ccle/) and 10 CML patients’ samples (five chronic phase and five blastic crisis samples [66]), by performing two PCAs. However, in the whole transcriptome, the gene expression profile of Ph-positive myeloid leukemia cell lines (Supplemental Fig. 8a) and that of CML patients’ samples (Supplemental Fig. 8b) were substantially different from that of p210 or p190 *BCR::ABL1* sublines and parental cells, regardless of whether cultured with GM-CSF or not. We also performed principal component analysis using the 16 genes upregulated by retroviral gene transfer of p210 *BCR::ABL1* fusion in HL-60 [67], an acute myeloid leukemia cell line, which includes *PIM1* oncogene, a signaling kinase (a guanine nucleotide exchange factor and Ras homolog) [68], *RAPGEF2*, a member of the RAS subfamily of GTPases that function in signal transduction [69], *HOXB2*, *SOX5*, and *KLF1*, transcription factors, and GAGE antigens, a family of cell surface antigens originally identified in melanoma cells. Of note, in these 16 genes, the gene expression profile of Ph-positive myeloid leukemia cell lines (Supplemental Fig. 8c) and that of CML patients’ samples (Supplemental Fig. 8d) were more similar to that of p210 or p190 *BCR::ABL1* sublines than that of parental TF-1 cells. These observations suggested that generation or gene transfer of *BCR::ABL1* fusion in human myeloid leukemia cell lines may affect gene expression involved in signal transduction and transcriptional regulation, which are upregulated in Ph-positive myeloid leukemia cell lines and CML patients’ samples, at least in part. Interestingly, although p210 BCR::ABL1 and p190 BCR::ABL1 induced factor-independent cell growth through similar signaling pathways, the gene expression profiles of the two sublines were distinctive. In particular, myeloid lineage-related genes, including *CD93* [70], *MPL* [71], *RARB* [72], and *MECOM* [73], were upregulated in the p210 BCR::ABL1 subline, compared to the p190 BCR::ABL1 subline. Moreover, most of these genes were downregulated by imatinib treatment in the p210 BCR::ABL1 subline but not in the p190 BCR::ABL1 subline. These observations suggest that our cell system might aid further understanding of differences in oncogenic activities between p210 BCR::ABL1 and p190 BCR::ABL1.

Based on our success in TF-1, we have also attempted to generate the *Bcr::Abl1* and *BCR::ABL1* fusions in the murine Ba/F3 line and in human hematopoietic stem cells purified from cord blood cells, respectively, using the same strategy. Since we have had no success in these two cellular systems, we speculated that TF-1 could have some advantages in generating the *BCR::ABL1* fusion with this strategy.

In summary, we demonstrated that double cleavages at the breakpoints of *BCR* and *ABL1* genes by the CRISPR/Cas9 system generate a balanced translocation that mimics the Ph chromosome in a human factor-dependent leukemia cell line, indicating that simultaneous DSBs at the *BCR* and *ABL1* breakpoints could be initiation events in Ph+ leukemia oncogenesis. Although the utility of the simultaneous introduction of the DSBs by the CRISPR/Cas9 system for the generation of *BCR::ABL1* fusion is limited to TF-1 cells thus far, our strategy may provide a novel platform for functional evaluation of the oncogenic activities of *BCR::ABL1* in the near future. (4421/4500 words).

## Supplementary information

Supplemental Figure Legends

Supplemental Fig 1

Supplemental Fig 2

Supplemental Fig 3

Supplemental Fig 4

Supplemental Fig 5

Supplemental Fig 6

Supplemental Fig 7

Supplement Table 1

Supplement Table 2

Supplement Table 3

Supplement Table 4

Supplement Table 5

Supplement Table 6

Supplement Table 7

## Data Availability

The sequence reads are available at the DDBJ Sequence Read Archive (DRA014097).
