# Supplementary material for: Creation of Philadelphia chromosome by CRISPR/Cas9-mediated double cleavages on BCR and ABL1 genes as a model for initial event in leukemogenesis
Source: Cancer Gene Ther. 2022 Aug 23;30(1):38–50. doi: 10.1038/s41417-022-00522-w (PMC9842507; doi:10.1038/s41417-022-00522-w)
Supplement: Supplementary file 1 — Supplemental Figure Legends [file 41417_2022_522_MOESM1_ESM.docx]

**Figure Legends**

Supplement Fig 1. Sequences of *BCR::ABL1* (a) and *ABL1::BCR* (b) fusion sites of genomic PCR products after TA-cloning in the p210 BCR::ABL1 sublines. Upper panel indicates original *BCR*and *ABL1* genomic sequences. Targeted PAM site is highlighted in orange. Arrows and arrowheads indicate sequences of sgRNA and Cas9 cleavage sites, respectively. Lower panel indicates alignment of sequences in eight clones. In each sequence, the variant nucleotide is indicated with an orange letter.

Supplement Fig 2. STR analysis of parental cells (upper panel) and the p210 *BCR::ABL1* subline (lower panel). Peaks corresponding to *ACTBP2*, *HGH*, *D3S1359*, and *D18S51* STRs are indicated.

Supplement Fig 3. Schematic representation of genomic location of FISH probes.

Supplement Fig 4. FISH analysis of parental cells (left panel) and p210 *BCR::ABL1*subline (right panel) in metaphase. Merged yellow signals for *BCR::ABL1* and/or reciprocal *ABL1::BCR* fusion genes are indicated by arrowheads.

Supplement Fig 5. Sequences of genomic PCR products for *BCR::ABL1* (a) and *ABL1::BCR* (b) fusion sites of genomic PCR products after TA-cloning in the p190 BCR::ABL1 sublines. Upper panel indicates original *BCR* and *ABL1* genomic sequences. Targeted PAM site is highlighted in orange. Arrows and arrowheads indicate sequences of sgRNA and Cas9 cleavage sites, respectively. Lower panel indicates alignment of sequences in seven clones. In each sequence, the variant nucleotide is indicated with an orange letter.

Supplement Fig 6. Heatmap of differentially regulated genes (FDR < 10^-13^) based on unsupervised hierarchical clustering in four samples: gray and green columns indicate parental cells cultured without and with GM-CSF, respectively; red and blue columns indicate p210 BCR::ABL1 and p190 BCR::ABL1 sublines cultured without GM-CSF, respectively. Red and blue color scaling indicates degrees of upregulation and downregulation to the mean expression across samples, respectively.

Supplement Fig 7. GO analysis of differentially expressed genes (FDR < 0.01, Log2 fold change < 1 or > -1) between p210 BCR::ABL1 and p190 BCR::ABL1 sublines (Supplement Table 7).

Supplement Fig 8. Comparisons of transcriptional profile between BCR::ABL1 sublines and Ph-positive myeloid leukemia cell lines or CML patients’ samples. Principal component analyses of the whole transcriptome (a, b) or expression of 16 genes, which are reported to be upregulated in HL-60 cell line by retroviral gene transfer of p210 *BCR::ABL1*fusion (c, d), were performed. Transcriptional profile of 59 myeloid leukemia cell lines, including 13 Ph-positive cell lines (orange) and 46 Ph-negative cell lines (light blue) (https://sites.broadinstitute.org/ccle/) (a, c), or five blastic crisis (orange) and five chronic phase (light blue) samples of CML patients ^66^ (b, d) were compared with that of parental TF-1 cells cultured in the presence (green) or absence (gray) of GM-CSF and the p210 BCR::ABL1 (red) and p190 BCR::ABL1(blue) sublines cultured in the absence of GM-CSF. Characteristics of the 16 genes are as follows: oncogene/tumor suppressor (*PIM1* and *TUSC3*), signaling (*RAPGEF2*, *PRPF4B*, and *RRAS2*), transcription factors (*SOX5*,*HOXB2*, and *KLF1*), cell surface antigens and adhesion proteins (*GAGE12F*, *GAGE12G*, and *CTNNA1*), nuclear proteins (*SEPTIN8*, *NUP214*, and *H2AC20*), and others (*PDLIM5* and *PLIN2*).^67^
