# Supplementary figures and images for: Creation of Philadelphia chromosome by CRISPR/Cas9-mediated double cleavages on BCR and ABL1 genes as a model for initial event in leukemogenesis
Source: Cancer Gene Ther. 2022 Aug 23;30(1):38–50. doi: 10.1038/s41417-022-00522-w (PMC9842507; doi:10.1038/s41417-022-00522-w)

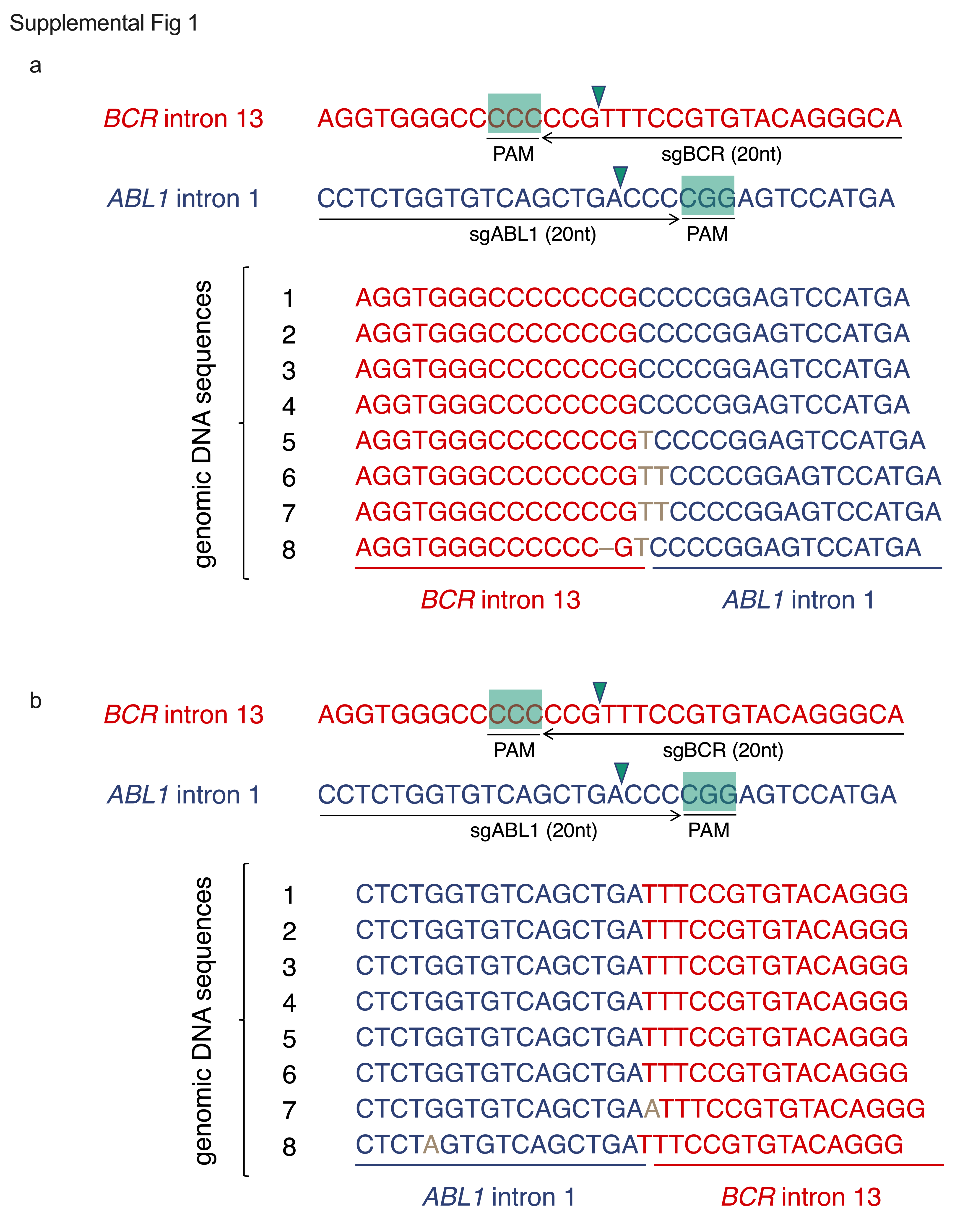

Supplement: Supplementary file 2 — Supplemental Fig 1 [file 41417_2022_522_MOESM2_ESM.tif]

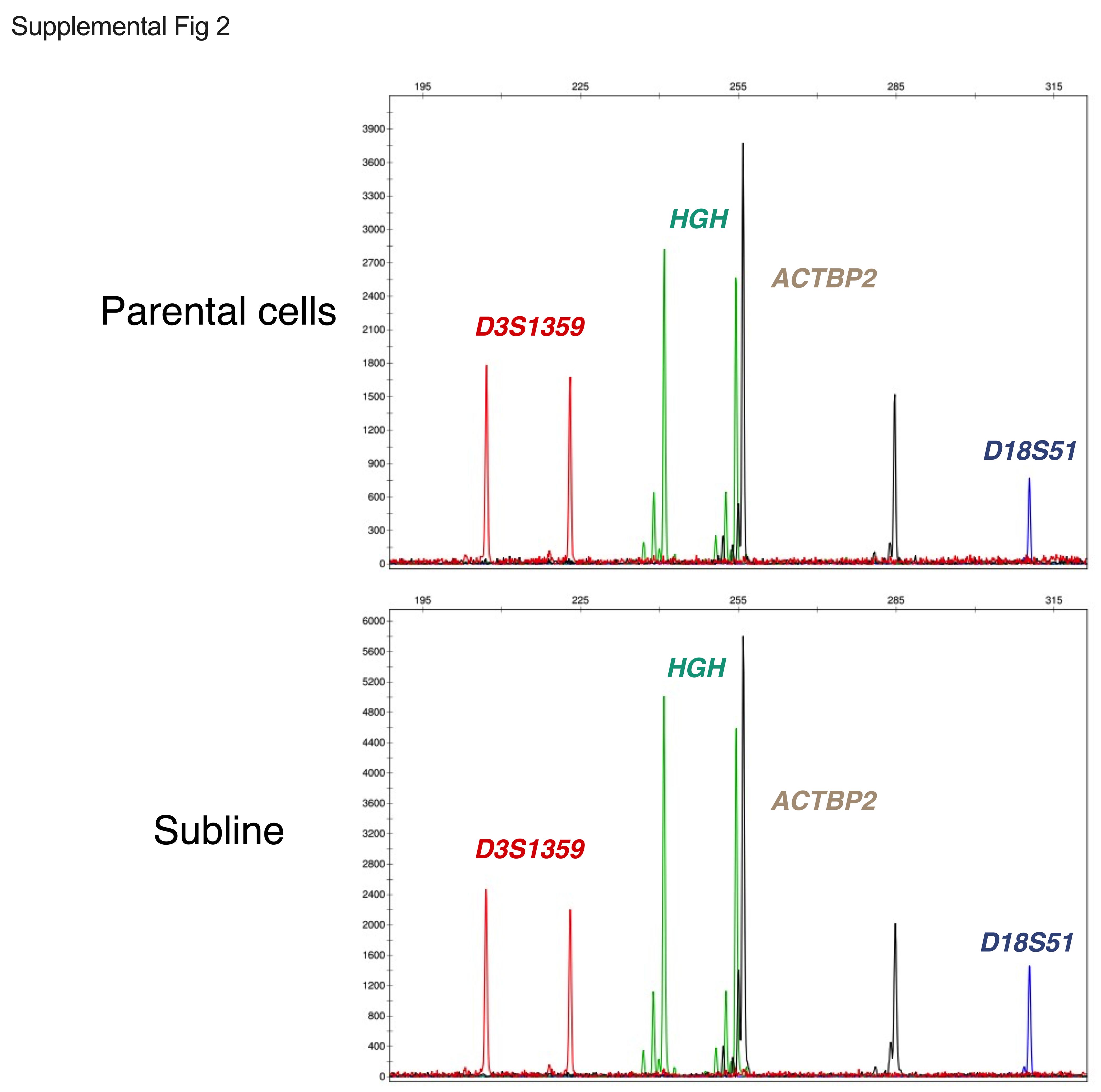

Supplement: Supplementary file 3 — Supplemental Fig 2 [file 41417_2022_522_MOESM3_ESM.tif]

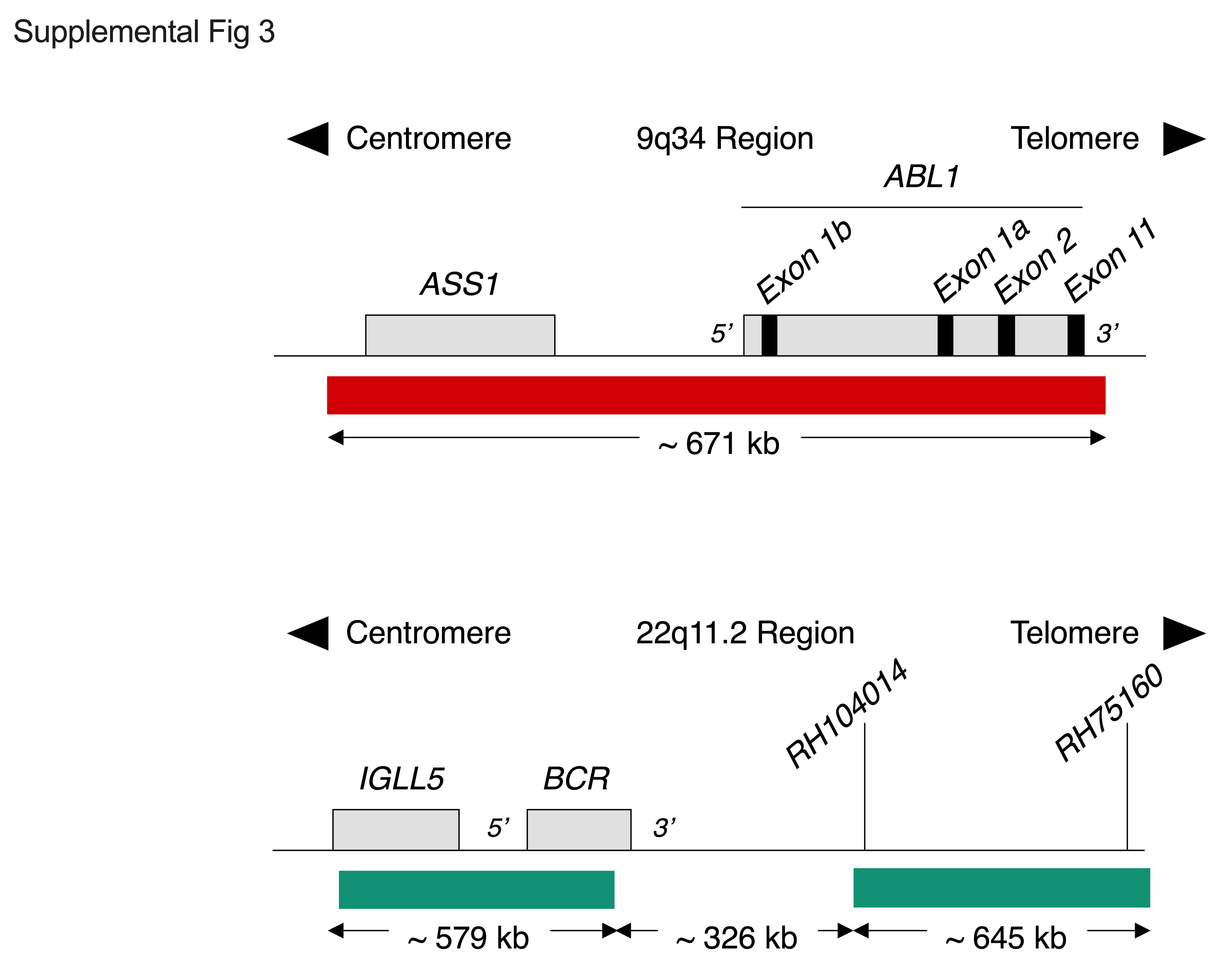

Supplement: Supplementary file 4 — Supplemental Fig 3 [file 41417_2022_522_MOESM4_ESM.tif]

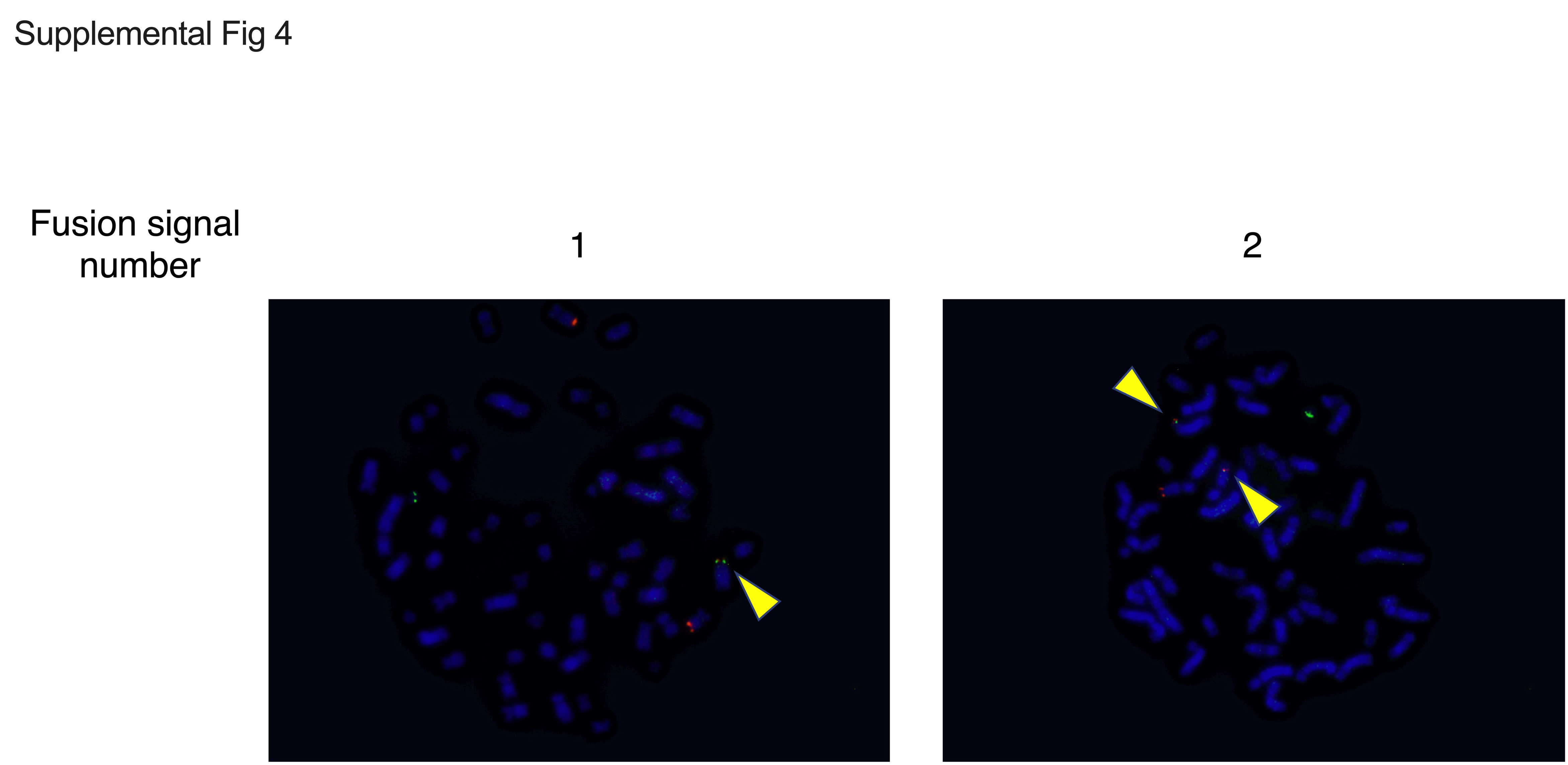

Supplement: Supplementary file 5 — Supplemental Fig 4 [file 41417_2022_522_MOESM5_ESM.tif]

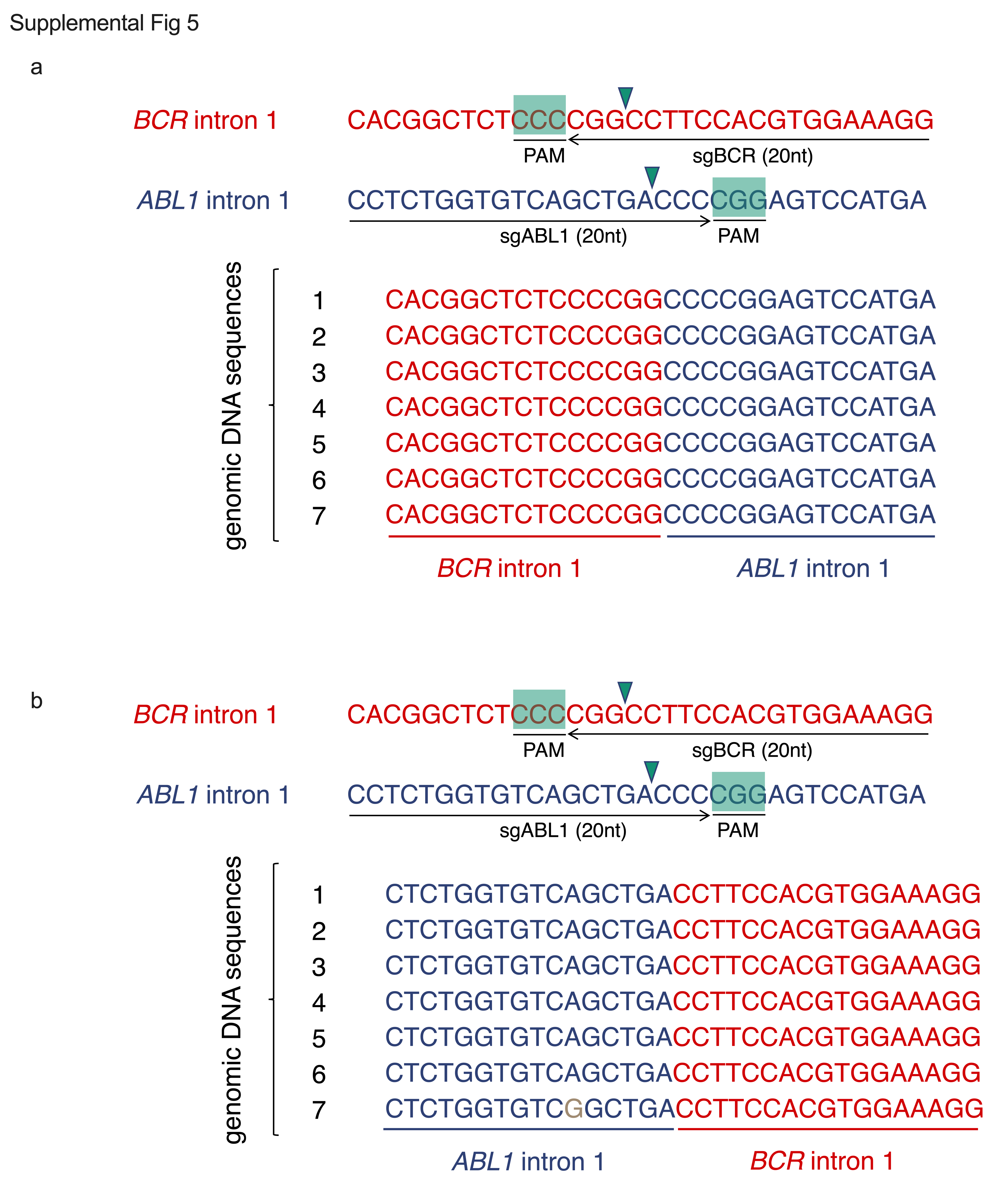

Supplement: Supplementary file 6 — Supplemental Fig 5 [file 41417_2022_522_MOESM6_ESM.tif]

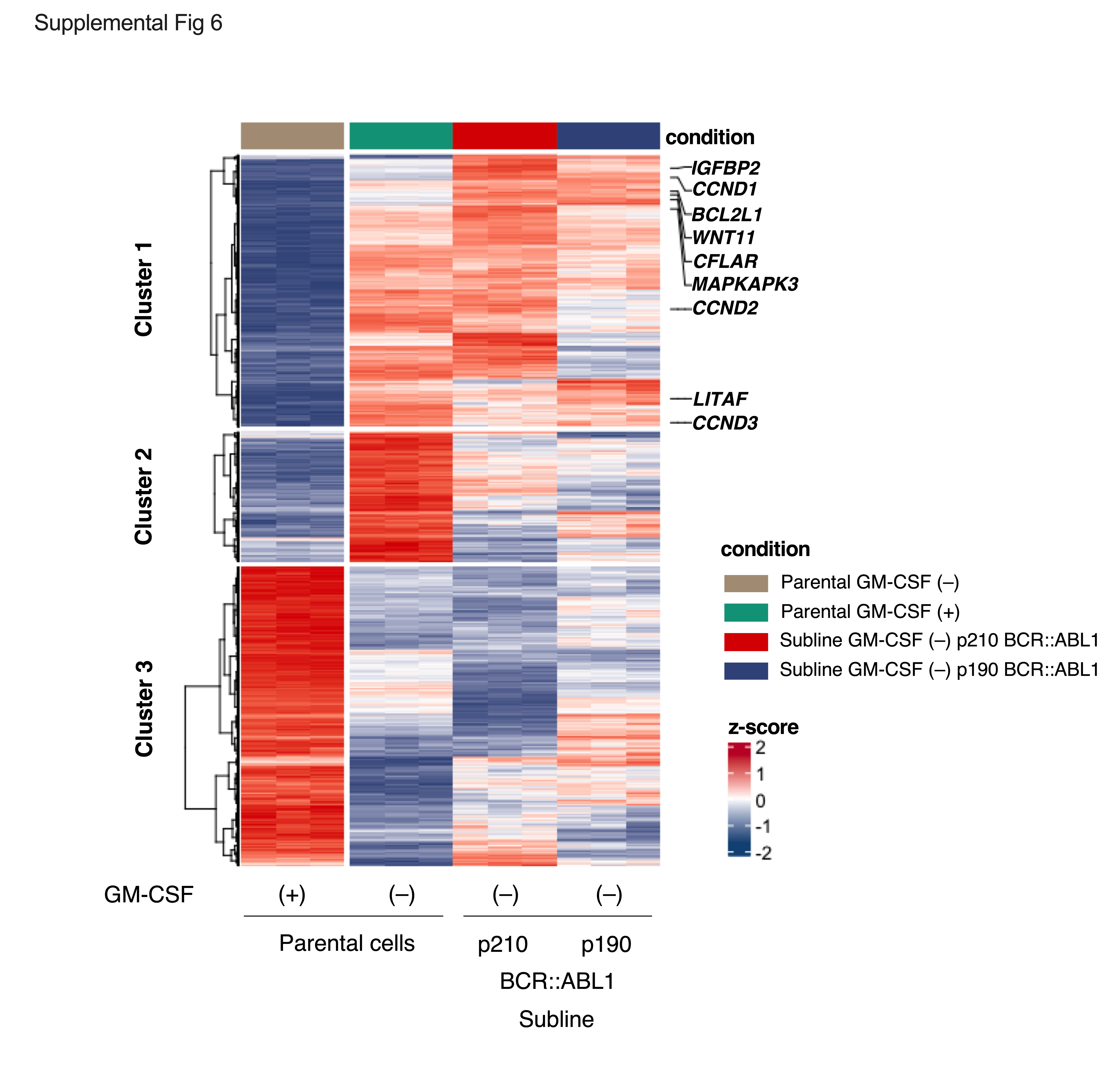

Supplement: Supplementary file 7 — Supplemental Fig 6 [file 41417_2022_522_MOESM7_ESM.tif]

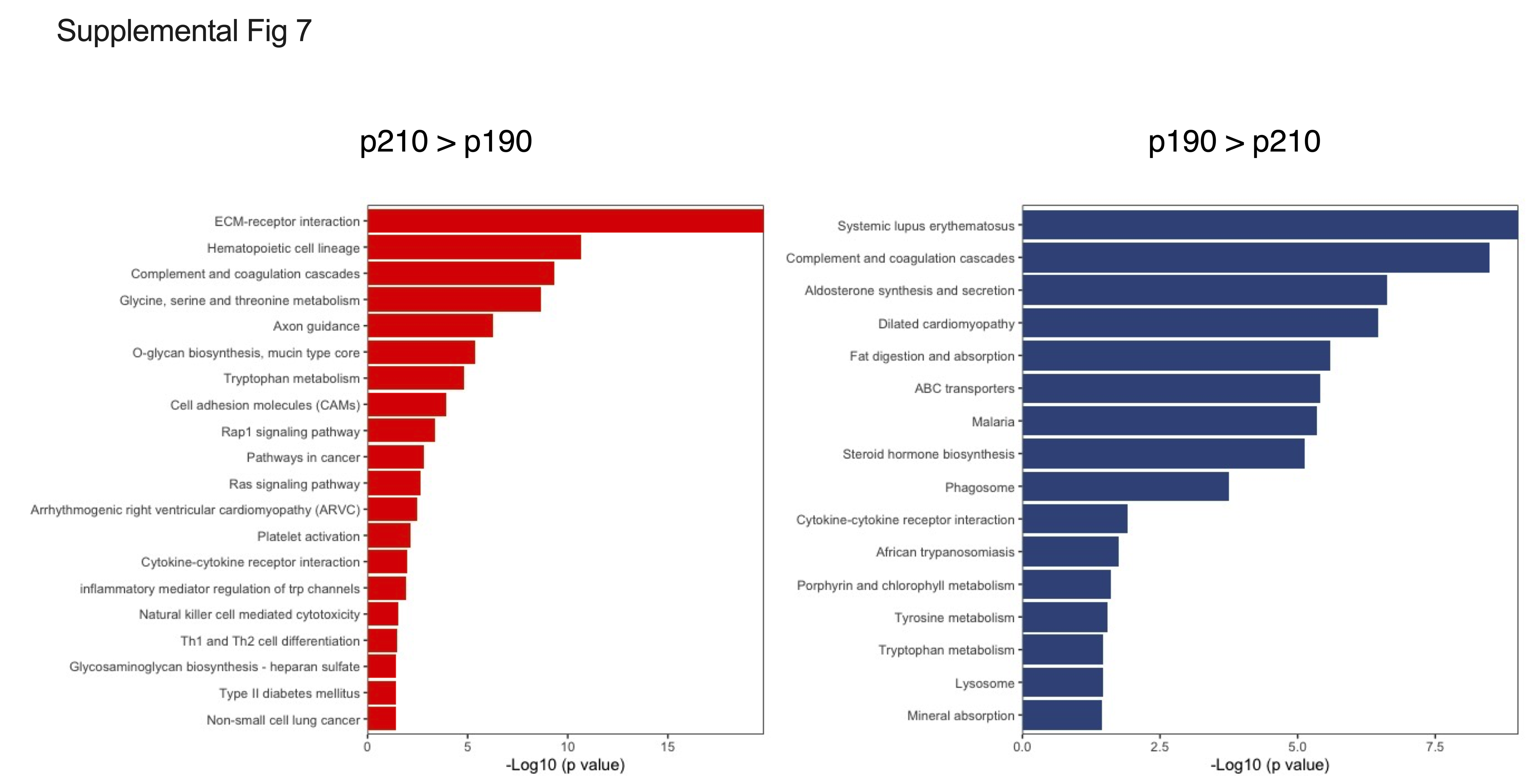

Supplement: Supplementary file 8 — Supplemental Fig 7 [file 41417_2022_522_MOESM8_ESM.tif]
